# The Morphologic Spectrum of Mediastinal Yolk Sac Tumors: Diagnostic Challenges and Pitfalls

**DOI:** 10.3390/cancers18071105

**Published:** 2026-03-29

**Authors:** Doaa Alqaidy, Cesar A. Moran

**Affiliations:** 1 Department of Pathology, King Abdulaziz University, Jeddah 22254, Saudi Arabia; 2 Department of Anatomical Pathology, The University of Texas MD Anderson Cancer Center, Houston, TX 77030, USA

**Keywords:** mediastinum, germ cell tumor, yolk sac tumor, morphologic patterns

## Abstract

Pure mediastinal yolk sac tumor is an uncommon and aggressive malignant germ cell neoplasm that presents considerable diagnostic difficulties owing to its pronounced clinical and morphological variability. Mediastinal yolk sac tumors, in contrast to their gonadal equivalents, typically occur at later stages, are typically associated with mixed germ cell components, and have a diverse array of histologic patterns that may resemble both germ cell and somatic malignancies. Accurate identification of these types of cancer is essential since diagnostic misclassification may significantly impact treatment and prognosis. This review provides a comprehensive overview of the morphologic spectrum of mediastinal yolk sac tumor, with emphasis on both classic and variant histologic patterns, including reticular, solid, glandular, papillary, hepatoid, and other less common growth forms. The immunohistochemical correlations of these patterns and their role in resolving diagnostic dilemmas are discussed, along with key differential diagnoses encountered in small mediastinal biopsy specimens. Particular attention is given to the limitations of limited tissue sampling, the impact of post-chemotherapy morphologic changes, and the potential for misinterpretation in this challenging anatomic site. By integrating morphologic features with clinical, radiologic, and laboratory findings, this review aims to enhance diagnostic accuracy and improve recognition of mediastinal yolk sac tumor across its diverse presentations.

## Simple Summary

Mediastinal yolk sac tumors are aggressive and diagnostically challenging due to their morphologic variability and frequent overlap with other malignancies. Accurate diagnosis relies on recognition of diverse histologic patterns, supported by immunohistochemistry and close clinicopathologic correlation, particularly in limited and post-treatment specimens. Small biopsies may fail to capture the full tumor spectrum, and therapy-induced changes can further obscure residual disease. Awareness of these pitfalls is essential to guide appropriate management and improve patient outcomes.

## 1. Introduction

Extragonadal germ cell tumor (GCT) is less frequent than its gonadal counterpart and presumably arises from the displacement of germ cell precursor cells during embryonic migration. Extragonadal germ cell tumors (GCTs) have been identified in anatomical areas including the central nervous system, mediastinum, retroperitoneum, and sacrococcygeal region; nevertheless, their total prevalence in these sites is rather low, constituting less than 10% of all GCT cases [1,2,3].

In the mediastinum, germ cell tumors have constituted around 1–15% of mediastinal tumors in adults and 25% in children across several series. One of the largest series on the incidence, classification, and staging of mediastinal germ cell tumors is the study by Suster and Moran in 1997, which examined 322 cases of primary mediastinal germ cell tumors [4]. Among their largest cohort, they indicated that mediastinal germ cell tumors have demographic and histopathological distributions similar to those of tumors found in the gonads. A significant result of this research is that MGCTs are more prevalent among young males. They indicated that teratomas and seminomas account for the majority of mediastinal GCT, with the other GCTs accounting for no more than 25% of the total [1,4]. In this study of non-seminomatous germ cell cancers, yolk sac tumors were the most prevalent, with a ratio of 6.1:1 [5].

Mediastinum yolk sac tumor (YST), also known as embryonal carcinoma infantile type or endodermal sinus tumor, is rare. It is more often seen as a component of mixed germ cell tumors rather than as a pure tumor. Teilmann et al. in 1967 reported a primary mediastinal YST in a 33-year-old male, which is considered the first description of such a tumor [6]. These tumors exhibited a higher incidence in the third and fourth decades of life, with only rare cases occurring in the second and fifth decades [7]. Few cases have been reported in infants as well.

For several reasons, surgical pathologists may find it difficult and challenging to diagnose this tumor in the mediastinum. First, YSTs exhibit a wide range of morphologic features, often resembling those of more prevalent tumors such as carcinomas. Furthermore, because of the rarity of this anatomical site, YSTs are not often included in the differential diagnosis of mediastinal lesions, which might delay patient care [1,7]. The diagnostic process becomes very complex, as tiny image-guided core needle biopsies serve as the initial samples for evaluating most mediastinal tumors. In this review, we will highlight some of those challenges that diagnostic surgical pathologists may encounter in the histopathological assessment of mediastinal yolk sac tumor, including a proposed diagnostic algorithm utilizing currently available immunohistochemical stains [8,9]. In the current literature, several studies have examined extragonadal germ cell tumors, but this study focuses on the morphologic variability and diagnostic challenges of primary mediastinal yolk sac tumors, especially in small biopsy specimens. Histologic patterns are compared to immunohistochemistry results and the relevant clinical context. This study highlights the whole morphologic range of mediastinal YSTs and their possible mimics to help surgical pathologists classify limited mediastinal specimens, which are often challenging.

## 2. Epidemiology and Clinical Features

The prepubertal testis is the most common site for pure yolk sac tumors (YSTs), whereas primary tumors in the mediastinum are relatively uncommon. Moreover, these tumors are predominantly observed in older populations, although YSTs can occasionally present in neonates. These tumors predominantly affect men; however, there are occasional reported cases in females [3,4].

Teilmann et al. were the first to describe a primary mediastinal YST in 1967 [6]. Later, in the largest series on primary mediastinal GCT to date, Moran et al. [4] reported on 64 non-teratomatous and non-seminomatous GCT, 38 of which were pure YSTs and 9 were mixed GCT with yolk sac components. Pure YST mostly affects male patients in their twenties and thirties and accounts for about 12% of all GCT in this area, as stated in their study.

Primary mediastinal yolk sac tumors are uncommon neoplasms mostly affecting young boys, usually manifesting in the second to fourth decades of life. They are a minor fraction of primary mediastinal germ cell tumors, representing around 1–4% of all germ cell tumors [10]. The prevalence of mediastinal germ cell tumors exhibits minor variations across populations, while extensive epidemiological data are lacking owing to the infrequency of the tumor. Certain research indicates possible regional and demographic disparities in occurrence; nevertheless, conclusive determinations of racial or genetic predisposition remain ambiguous. Continued accumulation of population-based data will be important to better understand potential demographic differences in disease distribution and outcomes.

Mediastinal YST, whether present alone or as part of a mixed GCT, has a dismal prognosis, particularly for individuals with advanced-stage cancers; nevertheless, patients whose illness is contained to the mediastinum and who undergo intensive chemotherapy may sometimes achieve long-term survival [11]. The clinical symptoms depend on the lesion’s size [9]. Patients with larger tumors exhibit symptoms associated with the compression of nearby mediastinal structures, while those with smaller tumors may remain asymptomatic [12].

Serum biomarkers are essential for the diagnosis and monitoring of mediastinal germ cell tumors, especially yolk sac tumors. Alpha-fetoprotein (AFP) serves as the most sensitive and specific biomarker for yolk sac tumors, often exhibiting significant elevation upon presentation, hence offering robust diagnostic support even in tiny or morphologically ambiguous biopsy samples [10,13]. Serial AFP readings are crucial for risk classification, evaluating therapy response, and the early identification of residual or recurrent illness. In contrast, β-human chorionic gonadotropin (β-hCG) is usually normal or only mildly elevated unless mixed germ cell components are present, while lactate dehydrogenase (LDH) serves as a nonspecific indicator of tumor burden [14,15].

Pure YST in individuals above the age of 15 has a favorable prognosis [13]. Additional characteristics correlated with clinical outcomes include age, tumor extent, volume of metastatic tumors, completeness of surgical excision, proportion of tumor necrosis post-chemotherapy, and significantly raised pre-treatment tumor markers [15,16].

Mediastinal yolk sac tumors are treated with multimodal approaches, mostly with platinum-based combination chemotherapy, including bleomycin, etoposide, and cisplatin (BEP). Serum alpha-fetoprotein levels are critical for evaluating therapy efficacy. Surgical excision of remaining mediastinal masses is advised, when possible, since viable tumor or teratomatous elements may remain post-chemotherapy [15]. Radiotherapy has a limited impact, and the overall prognosis is lower than that of gonadal yolk sac tumors [17].

## 3. Pathologic Features

### 3.1. Gross Pathology

**Figure 1:**
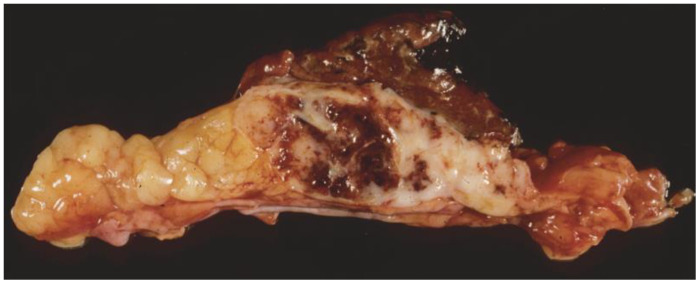
Mediastinal yolk sac tumor displaying a mass of tumor that is partly hemorrhagic and not encapsulated.

In general, there are no distinct gross features that facilitate the distinction of yolk sac tumor from other mediastinal germ cell tumors [3]. They often present as unencapsulated, soft-tissue masses that invade surrounding tissues, similar to other mediastinal tumors [9,13]. The tumors range in size from a few centimeters to 20 cm in maximum diameter, as documented in most studies, with areas of hemorrhage and/or necrosis (Figure 1).

### 3.2. Microscopic Features

The morphologic diversity of a yolk sac tumor, which often mimics more prevalent mediastinal tumors, such as carcinomas or sarcomas, is a problematic diagnostic feature of this tumor, as noted earlier [1,13]. These morphologic growth patterns pose major obstacles to the surgical pathologist during daily sign-out, as many cases fall outside their standard practice. Multiple growth patterns have been described for these tumors in the gonads, including reticular, vitelline, pseudo papillary, solid, myxomatous, sarcomatoid, macrocystic, hepatoid, and spindle cells or a combination of them. All histopathologic growth patterns are likewise observed in mediastinal yolk sac tumor. Another extremely rare characteristic of YST noticed in the mediastinum is the development of significant cystic changes reminiscent of those found in multilocular thymic cysts [3].

#### 3.2.1. Classic Morphologic Patterns

**Figure 2:**
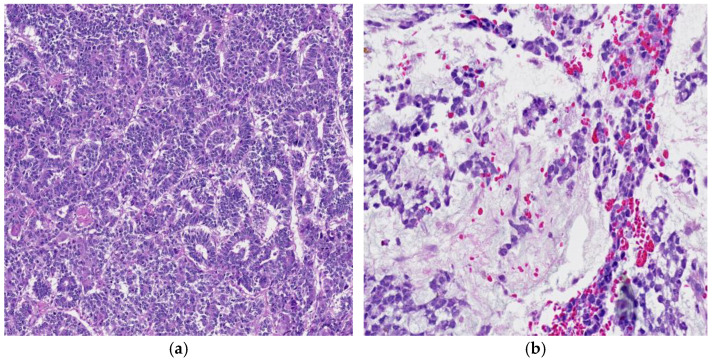
(**a**) The characteristics reticular/microcystic pattern of the yolk sac tumor, 10× H&E and (**b**) YST exhibiting significant edematous “myxoid” stroma.

The predominant morphology of these tumors is the reticular/microcystic pattern (Figure 2a), defined by areas featuring a loose meshwork of glandular or ductal structures lined by low columnar to flattened epithelial cells. The coalescence of these microcysts in certain regions resulted in the formation of macrocysts [9].

**Figure 3:**
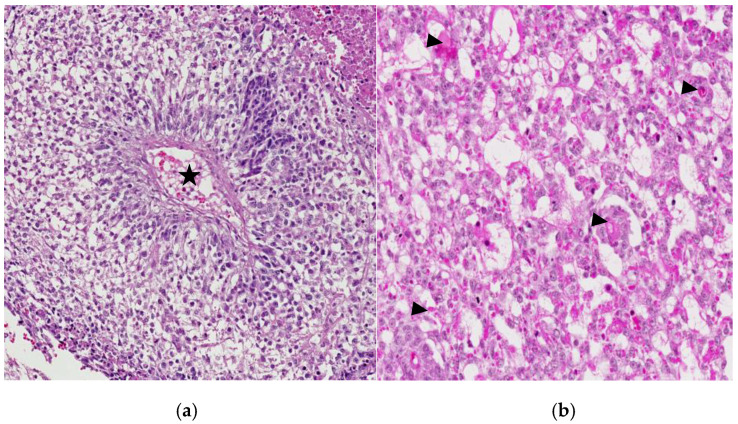
(**a**) Schiller–Duvall bodies are considered the characteristic morphologic feature of YST, 100× H&E and (**b**) at a higher magnification, a YST reveals many globules, arrowheads, that are translucent, 100× H&E.

The neoplastic cellular proliferation exhibited a lacelike growth pattern, characterized by the creation of many microcystic structures [14]. One characteristic morphology of this pattern is Schiller–Duvall bodies, composed of papillary projections lined by primitive epithelial cells, extending into microcystic spaces and containing a single central blood vessel. Schiller–Duvall bodies, together with intracytoplasmic and extracytoplasmic hyaline globules, are prevalent in this endodermal sinus pattern and are readily identifiable (Figure 3a). The identification of Schiller–Duval bodies, which are indicative of yolk sac tumors, will significantly enhance diagnostic accuracy. Hyaline globules that are diastase-resistant and highly eosinophilic are another hallmark of YST [7,11]. These globules may be seen intracellularly and distributed throughout the stroma. Evidence suggests that alpha-fetoprotein and other plasma protein aggregates contribute to these hyaline globules (Figure 3b).

**Figure 4:**
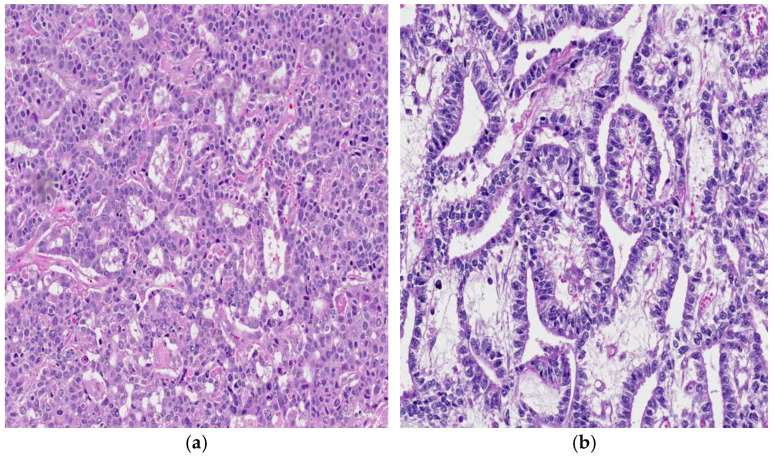
(**a**) Mediastinal yolk sac tumor displaying a glandular growth pattern, 60× H&E and (**b**) the characteristic vacuolated columnar cells of the yolk sac tumor resemble embryonic intestine architecture 100× H&E.

Glandular patterns may manifest as single glands, tubules, cysts, or papillae; sometimes, merged glands provide a cribriform or solid look (Figure 4a). Their unique cytological feature is apical and subnuclear cytoplasmic vacuolation, typical of the columnar lining of the embryonic intestine (Figure 4b). This histologic pattern, although it may be seen in mediastinal cases, is most commonly observed in ovarian YST [3].

**Figure 5:**
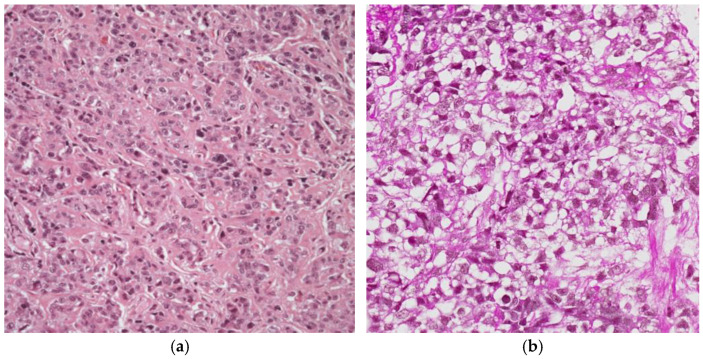
(**a**) YST with a solid growth pattern, 100× H&E and (**b**) the cells are polygonal with eosinophilic to clear cytoplasm, 100× H&E.

The solid form of YST is often characterized by a sheet-like arrangement of polygonal tumor cells with eosinophilic or clear cytoplasm and minimal pleomorphism (Figure 5). Since this solid form is similar to other mediastinal germ cell tumors, particularly seminoma, it may be difficult to distinguish it in tiny biopsies. In one study, cases from the testis, ovary, mediastinum, and sacrococcygeal area were included. A solid growth pattern constitutes about 86% of yolk sac tumors [14]. Diagnostic challenges arising from solid growths are mitigated by the common occurrence of more typical yolk sac neoplasia patterns nearby, making the identification of the tumor as a yolk sac tumor often straightforward. However, when solid foci are large, the tumor is entirely solid (as seen in 4% of our patients), or tumor sampling is limited, these situations pose diagnostic challenges [18].

**Figure 6:**
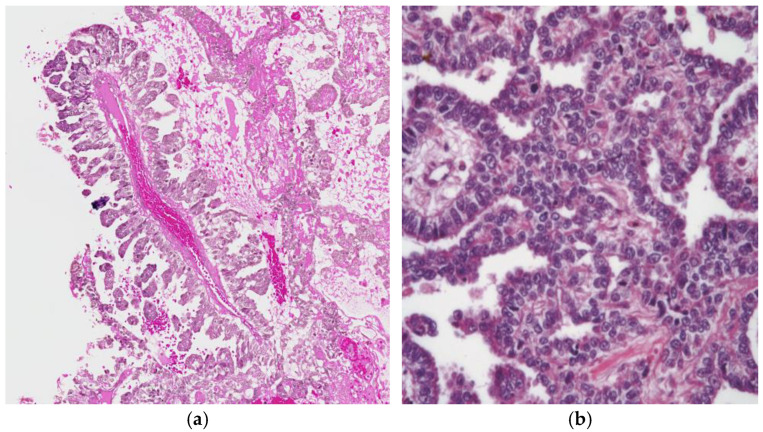
(**a**) YST with a papillary growth pattern, so-called intestinal pattern, 10× H&E and (**b**) the tumor cells developed papillary fronds, with or without fibrovascular centers, extending into cystic structures, 100× H&E.

A commonly observed morphological feature is the papillary growth pattern, characterized by irregular papillary fronds, with or without fibrovascular cores, spreading into cystic areas and surrounded by tumor cells that often have a hobnail appearance (Figure 6). The papillary growth pattern, referred to as the “intestinal” growth pattern, is characterized by neoplastic cells that form papillary structures resembling intestinal villi [11,13,14]. In some cases, the neoplastic cells may form pseudovascular spaces, mimicking a vascular tumor.

Mitotic figures were infrequent, and only minimal cellular atypia was seen. In various parts, the neoplastic cellular proliferation exhibited increased pleomorphic nuclear characteristics, including mitotic figures and sporadic giant, atypical tumor cells.

#### 3.2.2. Less Common and Unusual Morphologic Patterns

**Figure 7:**
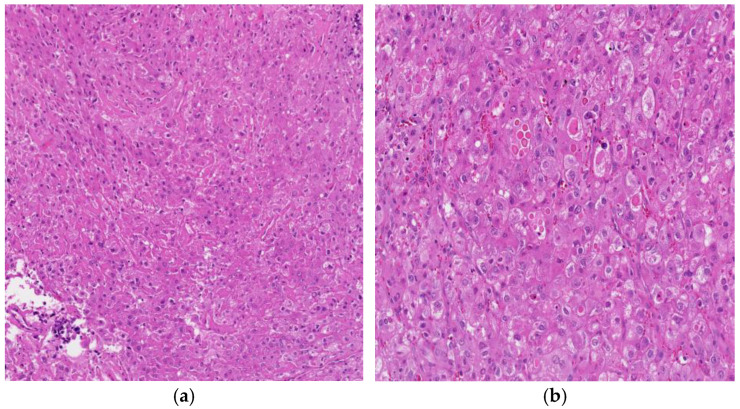
(**a**) YST displaying hepatoid morphology, 40× H&E and (**b**) the cells resemble hepatocytes by morphology, 100× H&E.

One of the unusual morphologic patterns of YST is the hepatoid pattern. This particular form of hepatic differentiation involves an arrangement in trabeculae that resembles normal liver tissue. The cells form clusters or sheets of tumor cells with richly eosinophilic cytoplasm (Figure 7). Cellular atypia and mitotic figures may also be seen in this growth pattern [19].

**Figure 8:**
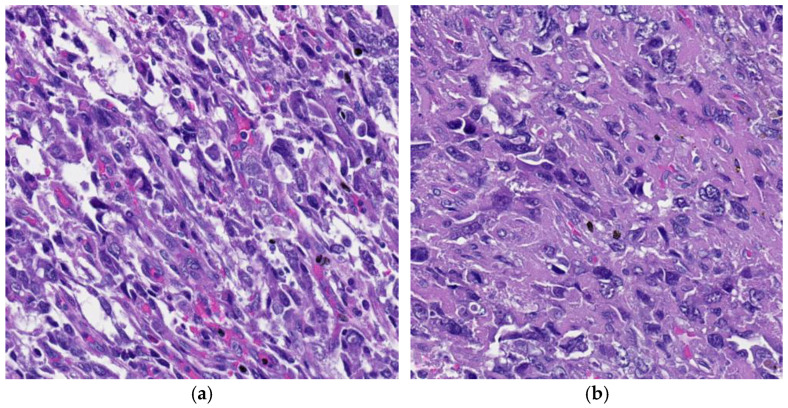
(**a**) YST displaying spindle cell morphology, 100× H&E and (**b**) the cells’ nuclei are elongated with focal bizarre morphology, 100× H&E.

An additional unusual morphologic variant of YST described in the mediastinum and may be a significant challenge for diagnosis, especially in tiny mediastinoscopic samples, is the spindle cell variant. In this histologic pattern, the cells are spindle-shaped with a somewhat storiform arrangement, suggestive of a fibrohistiocytic mesenchymal tumor (Figure 8). The spindle cells had elongated nuclei with a scattered chromatin pattern and inconspicuous nucleoli, along with sporadic mitotic figures. Some cases may exhibit myxoid matrix deposition [3,9,14].

#### 3.2.3. Chemotherapy-Induced Morphologic Changes

The evaluation of mediastinal yolk sac tumors post-chemotherapy poses additional diagnostic challenges due to treatment-induced morphological changes that may obscure or misidentify residual disease. Post-treatment specimens often exhibit significant necrosis, fibrosis, hemorrhage, and inflammatory infiltrates, which may overshadow the actual tumor cells during histologic examination [20]. The residual yolk sac tumor may present as localized lesions with changed architecture, characterized by simplified solid growth or cytologic atypia, hence complicating the identification of classic patterns such as reticular growth or Schiller–Duval bodies. Treatment effects may, in some instances, lead to apparent maturation or the appearance of somatic-type tumors, hence confusing interpretation [2].

Reliance on limited post-chemotherapy samples, therefore, carries a risk of underestimating residual viable tumor or misclassifying therapy-related changes as benign or non–germ cell processes. Careful sampling, judicious use of immunohistochemistry, and close correlation with pre-treatment histology and clinical findings are essential to avoid diagnostic pitfalls in this setting [11,20].

In post-treatment specimens, the immunophenotypic profile of yolk sac tumors is typically preserved, despite chemotherapy-induced morphologic alterations, including necrosis, fibrosis, and cytologic atypia. Residual tumor cells can be reliably identified using key diagnostic markers, such as AFP, glypican-3, and SALL4, despite occasional attenuated or focal staining [9].

While this review primarily focuses on YST, it is essential to note the presence of combined or mixed M-GCT. This category of malignancies includes several germ cell tumors (seminoma, yolk sac tumor, embryonal carcinoma, choriocarcinoma) devoid of teratomatous components. In our experience, these tumors account for approximately 5% of all M-GCT. Histopathological assessment is the definitive criterion for this specific diagnosis. Consequently, appropriate sampling of any mediastinal germ cell tumor is strongly recommended [7,11].

## 4. Immunohistochemistry and Molecular Characteristics

**Figure 9:**
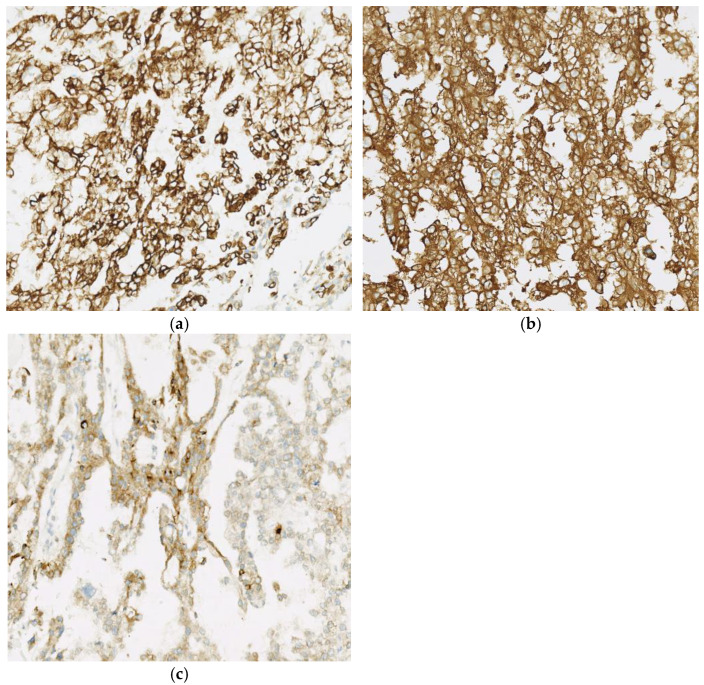
(**a**) YST showing diffuse keratin staining, 60× (**b**) AFP is considered a specific marker for YST and usually shows diffuse membranous staining, 60× and (**c**) glypican-3 expression is usually focal and patchy in most YST, 100 X.

Immunohistochemical staining plays a pivotal role in distinguishing yolk sac tumors (YST) from other germ cell neoplasms, particularly in challenging cases with overlapping morphologic features. Alpha-fetoprotein (AFP) is a highly sensitive and specific marker for YST, showing strong cytoplasmic positivity in most cases. Glypican-3 and SALL4 are also commonly expressed in YST, providing additional diagnostic support, especially when AFP staining is weak or equivocal. Other markers, such as cytokeratin (CK), PLAP, and CD117, may be variably expressed but are less specific (Figure 9). Importantly, negative staining for OCT4 and CD30 helps exclude seminoma and embryonal carcinoma, respectively [9,10].

PLAP reactivity is more variable in YST, although it is prevalent in testicular and mediastinal seminomas. The percentage of YST stained with PLAP ranges from 0% to 40% in mediastinal YST, 66% in extragonadal YST, and 25% to 85% in testicular YST. From very localized and weak staining to widespread and robust staining, this pattern has been shown in several prior studies to be inconsistent [15].

GATA-3 is a significant marker in surgical breast and bladder pathology. However, it is primarily associated with trophoblastic tumors within the spectrum of germ cell malignancies. Inconsistent findings regarding GATA-3 expression in testicular YST have been reported: Miettinen et al. identified GATA-3 in all 6 cases examined [21], but Osman et al. noted reactivity in just 3 of 25 cases (12%), and Liu et al. discovered no expression of this marker in 12 YST specimens [22]. In a study by Weissferdt et al., GATA-3 showed diffuse positivity in 5 of 14 patients (36%) with mediastinal YST [9].

The expression of CDX2 protein is often associated with cancers of the gastrointestinal tract and has recently been reported in testicular germ cell tumors (GCTs). Between 38% and 100% of mediastinal YST or tumors containing YST components have shown CDX2 expression [23]. One of the first markers used to diagnose GCT, CD30, is mainly linked to distinguishing embryonal tumors. A subgroup of YST may also be stained for CD30, with reported positive frequencies ranging from 0% to 57% [2,9,24].

Seminomas are the primary GCT tumor types that express c-kit (CD117), while embryonal carcinomas and teratomas also show some expression. Up to 60% of gonadal YST cases also showed c-kit reactivity. Compared with their testicular counterparts, we found that mediastinal YST expressed c-kit at approximately 21%. Finally, OCT3/4 protein is considered a specific and sensitive biomarker for intratubular germ cell neoplasia, seminoma, and embryonal carcinoma. This marker is frequently recognized as negative in gonadal and mediastinal YST [2,25].

YSTs of the mediastinum exhibit identical molecular changes as those seen at other locations, with isochromosome i(12p) serving as the diagnostic hallmark. Fluorescence in situ hybridization (FISH) and quantitative real-time polymerase chain reaction (qRT-PCR) are two methods that may identify these changes [26,27]. In a minority of cases, cytogenetic alterations can involve chromosomes 21 and X, as well as the loss of chromosome 13. Moreover, a significant incidence of aneuploidy is seen in YSTs from various sites, including mediastinal YST [26,28].

In general, primary mediastinal germ cell tumors, including YST, have a lower mutational load than that of gonadal GCTs. Prevalent mutations identified in PMGCTs include TP53 (46%), KIT (18%), KRAS (18%), PTEN (11%), NRAS (4%), and PIK3CA (4%) [28]. In the mediastinum, P53 mutations occur in up to 82% of YST cases and are related to resistance to cisplatin [13]. Advanced molecular diagnostics may help diagnose difficult cases of mediastinal YST. Next-generation sequencing, DNA methylation profiling, and AFP-mRNA detection can diagnose and monitor germ cell cancers [2]. These technologies are not yet commonly employed in clinical practice, although they may be effective in small biopsy specimens or post-treatment settings with restricted morphology.

The molecular landscape of mediastinal YSTs is incompletely described. Some of these pathways may have therapeutic significance, especially as targeted therapies evolve. Many mutations are unclear in terms of their clinical impact on tumor behavior, prognosis, and therapy response; thus, further research is needed.

## 5. Differential Diagnostic Considerations and Diagnostic Pitfalls

Diagnostic pitfalls can arise when interpreting immunohistochemistry in small biopsy specimens or in tumors with unusual morphologic patterns. Aberrant or focal expression of markers, sampling limitations, and technical issues may lead to misdiagnosis. Therefore, integrating morphologic assessment with a targeted immunohistochemical panel is essential for accurate diagnosis (Table 1). Awareness of these pitfalls and correlation with clinical and radiologic findings can further enhance diagnostic accuracy in routine practice [7].

In small mediastinal biopsies with suspected germ cell tumors, a stepwise immunohistochemical approach can facilitate accurate classification. Initial evaluation should include germ cell markers such as SALL4 and glypican-3, which are commonly expressed in yolk sac tumors. Additional markers, including AFP, may support the diagnosis, although staining can occasionally be focal or negative [2,24]. In cases where morphology suggests YST, but AFP is negative, the combined interpretation of SALL4 positivity, glypican-3 expression, and absence of OCT3/4 staining may still support the diagnosis. Correlation with serum AFP levels and radiologic findings is essential in such situations. This integrated approach is particularly valuable in limited tissue samples.

The reported expression rates of several immunohistochemical markers in yolk sac tumors, including PLAP, GATA-3, CDX2, CD30, and KIT, vary widely in the literature. These discrepancies likely reflect differences in study size, tumor heterogeneity, antibody clones, staining protocols, and interpretation criteria. Furthermore, the diverse morphologic patterns of YST may contribute to variable marker expression across different tumor components. Therefore, interpretation of immunohistochemical results should always be performed in conjunction with morphologic assessment and clinical context.

Accurate diagnosis of mediastinal yolk sac malignancies necessitates adequate tissue sampling. To enhance the likelihood of capturing diagnostic tumor architecture and ensure adequate tissue for immunohistochemical studies, it is recommended that multiple core biopsies be obtained whenever possible. Tissue preservation and resource allocation for ancillary studies are essential in the context of limited samples. Optimizing biopsy strategy and enhancing diagnostic yield can be achieved by fostering close communication among clinicians, radiologists, and pathologists.

### 5.1. Mediastinal Small Biopsy Challenges

The precise diagnosis and subclassification of mediastinal germ cell malignancies, especially non-seminomatous tumors and yolk sac tumors, are often hampered by the limitations of tiny mediastinal biopsy samples. These tumors often display significant intratumoral heterogeneity, characterized by the coexistence of numerous histologic components within the same mass. Small core biopsies may capture just one morphological pattern, perhaps overlooking localized yolk sac tumor elements or other non-seminomatous components [29]. Moreover, significant necrosis, bleeding, and fibrosis—characteristic of large mediastinal masses—may predominate in limited specimens, obscuring live tumors and resulting in nondiagnostic or misleading outcomes.

The broad morphologic spectrum of yolk sac tumor further complicates interpretation in small biopsies, as variant patterns such as solid, glandular, or hepatoid growth may closely mimic somatic malignancies. As a result, diagnosis often relies heavily on immunohistochemistry, which can be challenging in limited tissue due to variable or focal marker expression and immunophenotypic overlap with non–germ cell tumors. These limitations underscore the importance of close clinicopathologic correlation, including integration of radiologic findings and serum tumor markers such as alpha-fetoprotein [5]. In some cases, definitive classification may only be achieved on larger resection or post-chemotherapy specimens that allow more comprehensive sampling [2,3,30].

In complicated cases, an accurate diagnosis may be enhanced by multidisciplinary dialogue, consensus evaluation, and alignment with clinical and radiological data. Emerging tools, such as digital pathology platforms and telepathology consultation, may also facilitate expert review and improve diagnostic concordance in rare tumors.

### 5.2. Pattern-Based Pitfalls

The anterior mediastinum is a frequent location for metastatic tumors, making metastatic carcinoma from the gastrointestinal tract a significant issue since it may mimic glandular YST patterns. Furthermore, metastatic hepatocellular carcinoma, which may mimic the hepatoid pattern, should always be considered in the differential diagnosis. The presence of conventional YST areas and clinical history help distinguish YST from metastatic tumors [3]. Furthermore, based on our experience, doing a broad panel of immunohistochemical stains and understanding the possible staining patterns and pitfalls is a successful approach for achieving an accurate diagnosis [31]. Thymic carcinoma is another crucial differential diagnosis that can be mistaken for mediastinal YST; however, CD5 is often positive in thymic carcinoma when it is negative in mediastinal YST. Ultimately, YST exhibiting significant cystic alterations may mimic a multilocular thymic cyst; however, thorough sampling and meticulous investigation will identify YST cells, which are not present in multilocular thymic cysts [2,7].

The other germ cell tumors, such as seminoma, are a major differential for the solid variant of YST. Seminoma, on the other hand, is often positive for OCT3/4, c-kit, and D2-40, while being negative for glypican-3. Furthermore, embryonal carcinoma may share some architectural features with YST, which may lead to misdiagnosis. On the contrary, embryonal carcinoma cells often exhibit higher pleomorphism and are more likely to be positive for CD30 and OCT3/4 [4,32].

We should emphasize that the spindle cell variant of YST presents a significant diagnostic problem for general surgical pathologists. YST exhibiting spindle form may mimic a sarcoma, whether primary or secondary; however, the latter will lack conventional YST regions and will show negative staining for keratins while exhibiting positive staining for mesenchymal markers [33]. A significant consideration is the somatic-type sarcoma arising from mediastinal germ cell tumors (GCTs), which are comparatively less prevalent in the mediastinum than their gonadal counterparts. Most originate in conjunction with teratomas; however, research suggests that these malignancies may also originate from yolk sac tumors (YSTs). Sarcomas seem to manifest more commonly and at an earlier stage of the illness than adenocarcinoma, which generally presents later. The predominant histologic forms include rhabdomyosarcoma, primitive neuroectodermal tumors, and unclassifiable spindle cell sarcomas [34].

It is possible to misdiagnose a YST as a somatic-type sarcoma if it has a large amount of spindle cells. These neoplasms often exhibited spindled and epithelioid cells with a fibromyxoid stroma [35]. Because cytokeratin and SALL4 will be negative in the sarcoma but positive in the YST, immunohistochemical stains will be helpful in establishing the diagnosis. Given the aggressive clinical course of patients with somatic malignancy, it is recommended that extensive sampling and comprehensive histological investigation be performed to identify and definitively characterize these cases. Understanding the association and histological pattern is essential, since accurate categorization is vital and may ultimately influence the outcome and the prognosis [2,7,9].

## 6. Conclusions

A mediastinal yolk sac tumor is an uncommon and diagnostically challenging entity, noted for its significant clinical aggressiveness and significant morphological variation. As highlighted in this review, the histologic spectrum of yolk sac tumor is broad, often with multiple growth patterns coexisting within the same tumor, and this heterogeneity may be further accentuated in mediastinal presentations. The identification of both conventional and unusual morphological features, together with the appropriate use of immunohistochemical markers, is essential for precise diagnosis. Awareness of the potential for morphologic mimicry is particularly important in small biopsy specimens, where limited sampling may obscure the true nature of the tumor and complicate subclassification.

An integrated diagnostic approach that incorporates morphology, immunophenotype, clinical context, radiologic abnormalities, and serum tumor markers is required for the evaluation of suspected mediastinal yolk sac tumors. From a practical standpoint, meticulous pathological evaluation has immediate consequences for patient treatment, as precise categorization guides therapeutic decisions and prognostic stratification. Increased awareness of the whole morphologic range of mediastinal yolk sac tumors would improve diagnostic accuracy and lead to more consistent and successful patient management.

## Figures and Tables

**Table 1:** Immunohistochemical profile of mediastinal yolk sac tumor and key differential diagnoses.

Entity	SALL4	AFP	Glypican-3	OCT 3/4	CD30	PLAP	Cytokeratin	Other Helpful Markers
Yolk sac tumor	+(diffuse)	+(variable/focal)	+	−	−	−/focal	+	HepPar-1 (hepatoid, focal), CK8/18
Seminoma	+	−	−	+	−	+	−/focal	CD117, D2-40
Embryonal carcinoma	+	−	−	+	+	−	+	SOX2
Teratoma	−/focal	−	−	−	−	−	+	Linage-specific
Somatic-type adenocarcinoma	−	−	−	−	−	−	+	CEA, MOC31
Hepatocellular carcinoma (metastatic)	−	+	+	−	−	−	+	Arginase-1, HepPar-1
Thymic carcinoma	−	−	−	−	−	−	+	CD5, CD117

## Data Availability

The original contributions presented in this study are included in the article. Further inquiries can be directed to the corresponding author.
